# Successful management of VTE with essential thrombocythemia and cavernous transformation of the portal vein in early pregnancy: a case report

**DOI:** 10.1186/s12905-024-03051-w

**Published:** 2024-04-02

**Authors:** Xin Kang, Shibin Hong, Chengxi Tan, Wen Di, Ning Zhang

**Affiliations:** 1 Department of Obstetrics and Gynecology, Renji Hospital, School of Medicine, Shanghai Jiaotong University, Shanghai, China; 2 Shanghai Key Laboratory of Gynecologic Oncology, Renji Hospital, School of Medicine, Shanghai Jiaotong University, Shanghai, China; 3 Department of Ophthalmology, University of California, San Francisco, 1550 4th Street, San Francisco, CA 94143-2811 USA

**Keywords:** Cavernous transformation of the portal vein, Deep venous thrombosis, Essential thrombocythemia, Pregnancy, Case report, Anticoagulants

## Abstract

Due to the thrombohemorrhagic potential of essential thrombocythemia, pregnancy complicated by essential thrombocythemia should be recognized as a risk factor for obstetric complications. Here, we report the case of a patient with essential thrombocythemia with two significantly different pregnancy outcomes. Her first pregnancy (at 30 years of age) ended with an uneventful term delivery. However, the patient progressed to cavernous transformation of the portal vein in the period between her two pregnancies and subsequently experienced deep venous thrombosis during the first trimester of her second pregnancy (at 36 years of age). The patient’s platelet count during pregnancy was within the normal range, so she ignored previous instances of essential thrombocytosis (at 26 years of age). The patient’s main symptom was unrelieved pain in her leg. After that, she was successfully treated with anticoagulant throughout her entire pregnancy, resulting in a term vaginal delivery. This case highlights the importance of assessing pregnant patients with essential thrombocythemia according to their risk stratification. Specifically, risk assessments for potential pregnancy complications should take into account advanced maternal age and a previous history of thrombosis. Patients with essential thrombocythemia should be encouraged to participate in preconception counseling for risk assessment and to initiate prophylactic anticoagulation as soon as possible.

## Background

Essential thrombocythemia (ET) is a Philadelphia-negative chronic myeloproliferative neoplasm (MPN) characterized by increased risks of thrombotic and hemorrhagic complications [1]. 20% of ET patients are women of child-bearing age (traditionally 15 ∼ 45 years of age), and the prevalence of ET appears to be increasing [2]. There is a considerable risk of complications, including thrombosis, maternal hemorrhage, preeclampsia, and placental dysfunction, in pregnant women with ET, which may lead to fetal growth restriction (FGR), fetal loss or stillbirth. In conclusion, pregnancy complicated with ET warrants a multidisciplinary treatment approach. Emphasis should be given to early identification of high-risk pregnant women [3].

Here, we report the case of a 36-year-old multigravida patient with ET who had two pregnancies with significantly different outcomes. Her first pregnancy was uneventful, ending with a term delivery. However, the patient progressed to cavernous transformation of the portal vein (CTPV) in the period between her two pregnancies, after which she experienced deep venous thrombosis (DVT) during the first trimester of her second pregnancy. She was admitted to our hospital and was successfully treated with anticoagulant throughout the pregnancy, ending with a term vaginal delivery. The case presented here promoted our awareness of comprehensive risk stratification for patients with pregnancy complicated with ET. Specifically, advanced maternal age and a previous history of any thrombosis event increases the risk of pregnancy complications.

## Case presentation

A 36-year-old woman, gravida 2, para 1, at 12 weeks gestation with no history of miscarriage was urgently admitted to our emergency department due to unrelieved pain in her legs before her first prenatal examination. Her vital signs were stable: a blood pressure of 123/70 mmHg, a heart rate (HR) of 75 bpm, a respiratory rate (RR) of 20 bpm, and an oxygen saturation (SpO_2_) of 96% on room air. On initial assessment, her white blood cell count was 3.70 × 10^9^/L, her hemoglobin level was 100 g/L, her platelet count was 109 × 10^9^/L, and her D-dimer level was 1.52 mg/L (normal range: 0.8–11.6 mg/L). Emergency bilateral lower extremity vein ultrasonography was quickly performed and revealed deep vein thrombosis in her left lower extremity (incomplete occlusion of the common femoral vein, complete occlusion of the superficial femoral and popliteal veins). Obstetric ultrasonography revealed an intrauterine live fetus consistent with 12 weeks of gestation. The patient denied a history of dyspnea, chest distress, palpitations, abdominal pain, or vaginal bleeding. The patient denied a history of previous disease. Therefore, we made a preliminary diagnosis of early pregnancy complicated with acute lower extremity deep venous thrombosis (DVT).

**Fig. 1:**
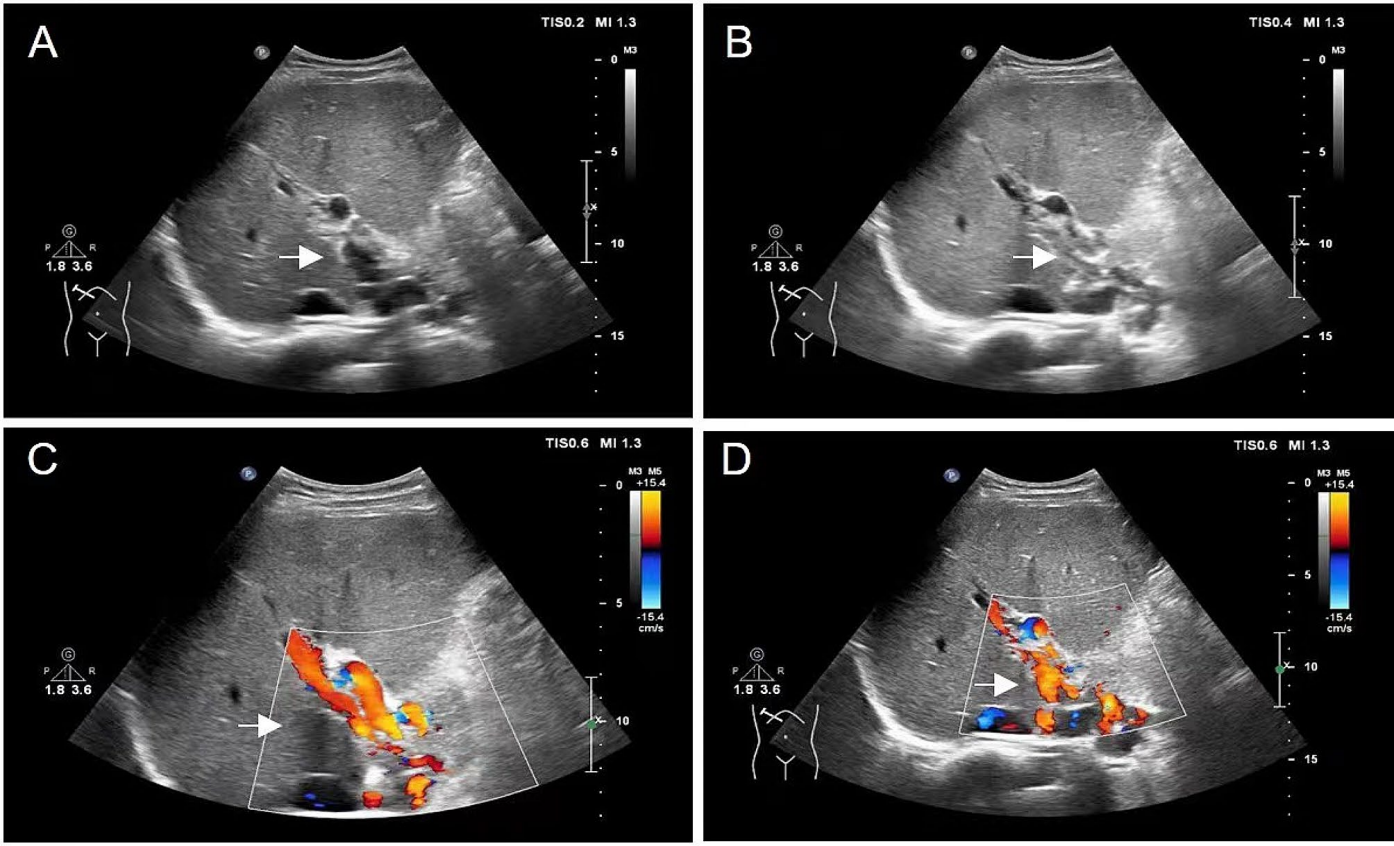
Ultrasonography for the identification of cavernous transformation of the portal vein (CTPV). (**A, B**) Doppler color flow imaging (DCFI) showing many spot- and slice-style color blood flow signals that are characteristic of CTPV. (**C, D**) The anechoic area in the first porta hepatis shows dilated vessels

Further laboratory tests revealed that the patient’s hepatic and renal function, thyroid function, serum electrolyte levels, and cardiac function were normal. Abdominal ultrasonography revealed a normal size and echotexture of the liver, the main portal vein at the first hepatic portal was not visible, and multiple collateral circulations had formed. Massive splenomegaly was also observed, and the lower edge of the spleen reached the umbilical level on heterogeneous internal echo (Fig. 1). The patient denied any history of upper gastrointestinal tract bleeding. Overall, based on the patient’s history of an unexplained occurrence of DVT and abnormalities on abdominal ultrasonography, we performed further active communication with the patient, and a more detailed medical history was revealed. The patient reported a so-called “cured” hematological disease, and the details are as follows:

### History of hematological diseases

In 2012, a physical examination revealed that the patient’s platelet count was elevated to 800 × 10^9^/L. She did not show any symptoms of fever, abdominal pain, diarrhea, nausea, or vomiting. After a bone marrow biopsy, the diagnosis of “primary thrombocytosis” was confirmed. She was treated with hydroxyurea and interferon for 1 ∼ 2 months. A decrease in her platelet count was observed (decreased to 400 × 109/L), so she stopped the treatment without regular follow-up. In 2018, she was admitted to the hospital with complaints of abdominal pain without definite causes, and her mandibular lymph nodes were significantly enlarged with pressure pain. Abdominal computed tomography (CT) revealed that the spleen was enlarged and that the blood vessels in the retroperitoneal cavity were twisted. Her platelet count was 263 × 10^9^/L, and her liver and renal function were within normal limits. A bone biopsy, as well as flow cytometry and genetic tests, was carried out to confirm the diagnosis of essential thrombocythemia (ET) with the V617F mutation of the Janus kinase 2 (JAK2) gene. Then, she was treated with the targeted pharmacological therapy of lucotinib (200 mg bid), and after one year, she discontinued treatment, with a platelet count of 200 ∼ 300 × 10^9^/L. Until her pregnancy in 2021, she had not returned for regular follow-up and never sought medical advice when preparing for this pregnancy due to her uneventful pregnancy in 2016. The patient felt that there was no association between the so-called “cured” hematological disease and her pregnancy outcome.

### Treatment and pregnancy outcomes

Based on the detailed medical history and a comprehensive evaluation, we considered that her thrombosis in early pregnancy was probably associated with ET, which is characterized by an increased risk of thrombosis, so anticoagulation was immediately initiated with body-weight-adjusted subcutaneous low-molecular-weight heparin (LMWH), specifically 4100 IU every 12 h. Additionally, her lower extremity was kept still in the acute stage. Two weeks later, when the patient’s symptoms were relieved and a repeat ultrasonography showed no progression of the thrombosis, she was discharged and remained on LMWH anticoagulant. She was closely followed up during her prenatal examinations. The CBCs showed that the platelet counts ranged from 97 ∼ 128 × 10^9^/L, and the D-dimer levels ranged from 0.61 ∼ 4.61 mg/L. Regular vessel ultrasonography showed that the thrombus in her left lower extremity had stabilized. During the second, and especially the third, trimester, obstetric ultrasonography was scheduled regularly to closely monitor the growth of her fetus and to evaluate whether there were any signs of fetal growth restriction (FGR). Fortunately, the patient maintained good compliance with the LMWH anticoagulant twice per day and followed a regular prenatal examination plan. Her condition remained stable, and the growth of her fetus matched well with the gestational age.

**Fig. 2:**
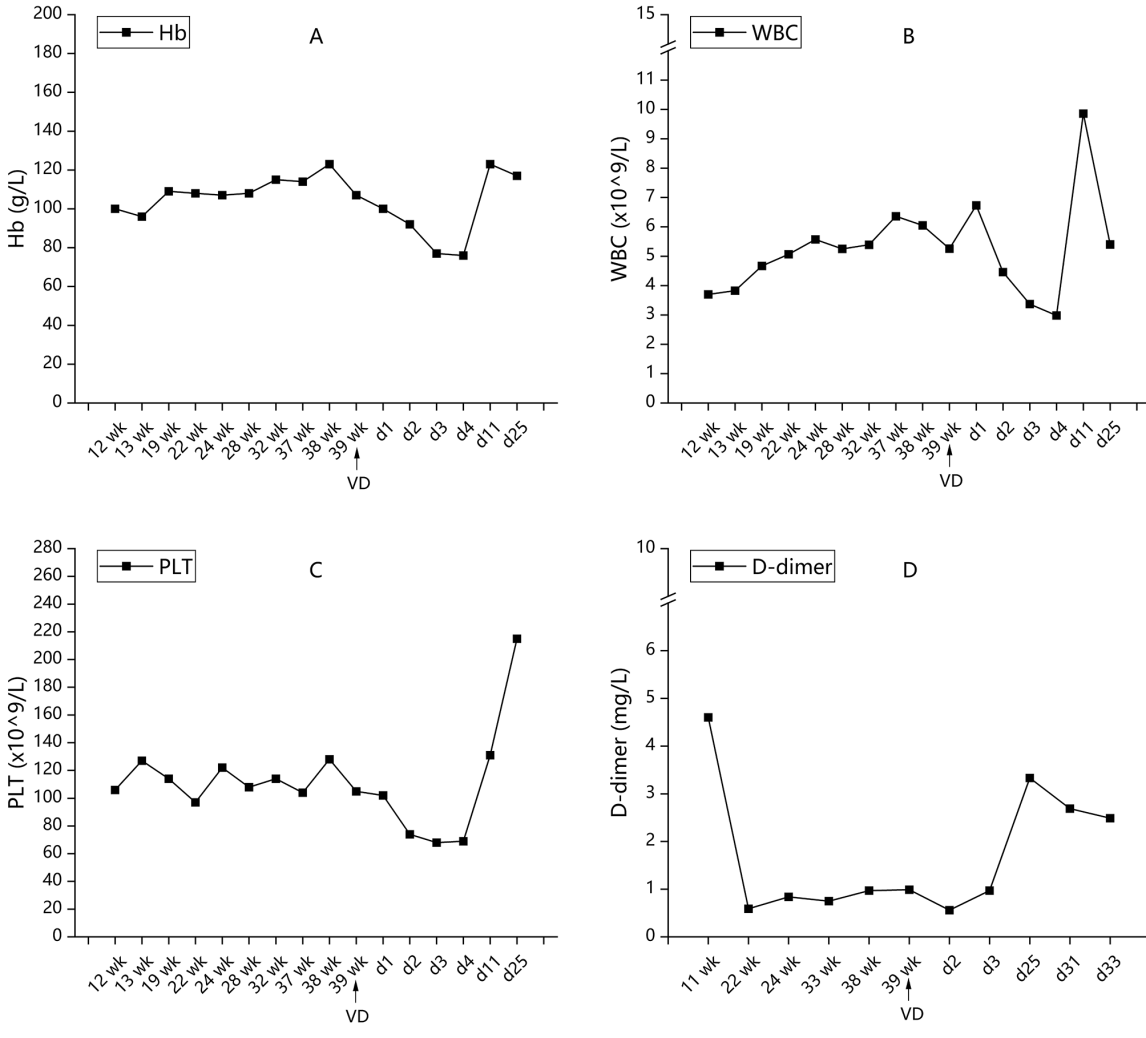
Changes in relevant clinical indicators in the antepartum period and puerperium period. The (**A**) platelet count, (**B**) hemoglobin level, and (**C**) white blood cell (WBC) count were relatively stable during the antepartum period, with acceptable fluctuations in the perinatal and puerperium stages. (**D**) D-dimer levels peaked when thrombosis first occurred but were controlled immediately after admission and remained within the normal range during the remainder of the pregnancy. Only a slight increase was observed postpartum during the period of laparoscopic myomectomy. VD: vaginal delivery

Neither bleeding events nor new thrombotic events were observed during prenatal examinations. At 39 weeks of gestation, she experienced spontaneous labor as expected, ending with an uneventful vaginal delivery, and gave birth to a 3960 g baby girl with an Apgar score of 10 (1 min) and − 10 (5 min). The newborn’s routine blood workup revealed a platelet count of 115 × 10^9^/L. Within several days postdelivery, the patient experienced a transient decrease in the platelet count, with the lowest decrease of 65 × 10^9^/L, but it soon returned to normal (131 × 10^9^/L). She was then discharged and remained on an anticoagulant with LMWH. At the 3-month follow-up, her overall condition was satisfactory, and her baby was healthy. Figure 2 shows the changes in the patient’s platelet count, hemoglobin level, white blood cell (WBC) count and D-dimer level in the antepartum and puerperium periods.

## Discussion

It has been demonstrated that pregnancy complicated with ET should be recognized as an issue because it is associated with an increased risk of obstetric complications due to its thrombohemorrhagic potential. The expert consensus recommends that decision-making should be based on risk stratification in regard to antithrombotic therapy, but how to define high-risk pregnancies and provide a possible step-up therapy for patients at risk is still largely unknown. Our report describes a patient with ET who experienced two pregnancies with significantly different outcomes, which indicated that advanced maternal age and a previous history of thrombosis increases the risk of pregnancy complications.

Our patient was diagnosed with ET according to the revised 2016 World Health Organization (WHO) criteria [4]. The unexpectedly elevated PLT count (800 × 10^9^/L) during regular check-ups caught the patient’s attention and led her to seek further professional evaluation. However, after cytoreduction therapy, similar to our present case involving hydroxyurea, interferon, or targeted pharmacological therapy with lucotinib, the recovery of the PLT count to within the normal range might cause the underlying ET condition to be easily neglected by obstetricians. In our case, the patient presented with an unexplained occurrence of DVT at 12 weeks gestation, and screening for thrombophilia and all coagulation factors, as well as for autoimmune diseases, were negative. The patient believed that the “cured” disease would have no impact on her pregnancy, especially given her past history of an uneventful term pregnancy 6 years ago in her 30s. However, several risk factors changed between her two pregnancies. First, in 2018, in the period between the two pregnancies, abdominal CT revealed dilated and twisted collateral vessels in the patient’s abdominal cavity, which indicated the possibility of portal vein thrombosis. Transformation of the portal vein (CTPV) is a rare and incurable complication of portal vein thrombosis (PVT) and should be interpreted as a concerning sign of ET exacerbation. An international study of 891 ET patients suggested that a history of thrombosis is an independent risk factor for predicting future vascular events (HR = 1.9) [5]. Thus, patients should be classified as “high risk” according to the International Prognostic Score of Thrombosis (IPSET-thrombosis) [5]. Second, our patient was 36 years old at the time of her second pregnancy, while her first pregnancy was at 30 years of age. An advanced maternal age greater than 35 years increases the risk of antenatal VTE by 1.6- to 2-fold; thus, according to the 2015 RCOQ guidelines [6], a maternal age older than 35 years should arouse public concern regarding the increased risk of thrombosis. Therefore, all of these factors resulted in different risk factors between her two pregnancies. As soon as the diagnosis of DVT was confirmed, we started anticoagulant therapy with 4100 IU LMWH twice per day until the day before delivery. In our case, we maintained the patient with LMWH throughout the entire puerperium, which paved the way for an uneventful puerperium.

Although most reported patients with ET have platelet counts that are much greater than normal, the platelet counts in our patient throughout pregnancy remained stable and within normal ranges. However, our patient still progressed to VTE in this context, which indicated that the platelet count did not predict the ET pregnancy outcome. This conclusion was supported by a number of studies from other groups as well [7–9]. Wright CA et al. studied 43 pregnancies that occurred in 20 ET patients, in which the platelet count at conception ranged from 400 to 2500 × 10^9^/L, and concluded that the PLT count either at diagnosis or at conception did not predict pregnancy outcomes in patients with ET [10].

The case presented here will promote awareness of risk stratification for patients with pregnancy complicated by ET, advanced maternal age and a previous history of thrombosis, which should strengthen the risk evaluation of pregnancy complications. ET patients should be encouraged to participate in preconception counseling for risk evaluation and prompt initiation of prophylactic anticoagulation.

## Data Availability

The relevant materials used in the present study are available from the corresponding author upon reasonable request.

## References

[1] Tefferi A, Vardiman JW (2008). Classification and diagnosis of myeloproliferative neoplasms: the 2008 World Health Organization criteria and point-of-care diagnostic algorithms. Leukemia.

[2] James C, Ugo V, Le Couédic JP, Staerk J, Delhommeau F, Lacout C (2005). A unique clonal JAK2 mutation leading to constitutive signalling causes polycythaemia vera. Nature.

[3] Burbury K, Panigrahi A (2021). Esssential thrombocythaemia and pregnancy-A need for prospective study and a consensus on its management. Leuk Res.

[4] Arber DA, Orazi A, Hasserjian R, Thiele J, Borowitz MJ, Le Beau MM (2016). The 2016 revision to the World Health Organization classification of myeloid neoplasms and acute leukemia. Blood.

[5] Barbui T, Finazzi G, Carobbio A, Thiele J, Passamonti F, Rumi E (2012). Development and validation of an International Prognostic score of thrombosis in World Health Organization-essential thrombocythemia (IPSET-thrombosis). Blood.

[6] RCOG. Thromboembolic disease in pregnancy and the puerperium: acute management. Green-Top Guideline. 2015 2015(No. 37b, RCOG, London).

[7] Passamonti F, Randi ML, Rumi E, Pungolino E, Elena C, Pietra D (2007). Increased risk of pregnancy complications in patients with essential thrombocythemia carrying the JAK2 (617V > F) mutation. Blood.

[8] Gangat N, Wolanskyj AP, Schwager S, Tefferi A (2009). Predictors of pregnancy outcome in essential thrombocythemia: a single institution study of 63 pregnancies. Eur J Haematol.

[9] Melillo L, Tieghi A, Candoni A, Radaelli F, Ciancia R, Specchia G (2009). Outcome of 122 pregnancies in essential thrombocythemia patients: a report from the Italian registry. Am J Hematol.

[10] Wright CA, Tefferi A (2001). A single institutional experience with 43 pregnancies in essential thrombocythemia. Eur J Haematol.

